# Analysis of Hepatic Lipid Metabolism and Immune Function During the Development of Collagen-Induced Arthritis

**DOI:** 10.3389/fimmu.2022.901697

**Published:** 2022-06-16

**Authors:** Yingjie Shi, Jun Shu, Zhangchi Ning, Dancai Fan, Haiyang Shu, Hanxiao Zhao, Li Li, Ning Zhao, Cheng Lu, Aiping Lu, Xiaojuan He

**Affiliations:** ^1^ Institute of Basic Research in Clinical Medicine, China Academy of Chinese Medical Sciences, Beijing, China; ^2^ Shanghai Innovation Center of TCM Health Service, Shanghai University of Traditional Chinese Medicine, Shanghai, China; ^3^ Institute of Clinical Medical Science, China-Japan Friendship Hospital, Beijing, China; ^4^ Institute of Basic Theory for Chinese Medicine, China Academy of Chinese Medical Sciences, Beijing, China; ^5^ The Second Clinical Medical College of Guangzhou University of Chinese Medicine, Guangzhou, China; ^6^ Law Sau Fai Institute for Advancing Translational Medicine in Bone and Joint Diseases, School of Chinese Medicine, Hong Kong Baptist University, Hong Kong, Hong Kong SAR, China; ^7^ Shanghai GuangHua Hospital of Integrated Traditional Chinese and Western Medicine, Institute of Arthritis Research, Shanghai Academy of Chinese Medical Sciences, Shanghai, China

**Keywords:** rheumatoid arthritis, liver, proteomics, lipid metabolism, immune function

## Abstract

The liver is essential for metabolic and immune functions and has been linked to systemic inflammatory diseases. However, the role of the liver is still elusive during the development of rheumatoid arthritis (RA), although there have been indeed some reports. We used label-free quantitative proteomics and experimental verification in this study to reveal the hepatic lipid metabolism and immune function during collagen-induced arthritis (CIA) development. The proteomics results revealed that the role of the liver differs in different phases of CIA rats. In terms of specific performance, hepatic lipid metabolism, which is primarily concerned with cholesterol, triacylglycerol, and phospholipid, was significantly influenced in the CIA induction phase, whereas the immune function, which includes binding of granulocytes, adhesion of immune cells, etc., was affected considerably at the peak phase of CIA rats compared to normal rats. Finally, the hepatic dynamic changes in CIA rats were further confirmed using targeted metabolomics and ELISA. We found that most fatty acids of the liver in the CIA induction phase were significantly decreased, and proteins related to complement activation and migration or adhesion of immune cells including C3, MMP-8, CTSZ, and S100A9 were significantly increased in the liver of CIA rats in the peak phase. Our findings indicated that the lipid metabolism and immune function of the liver were influenced in CIA rats. Thus, the conditions of the liver during RA development should be considered in therapeutic and nutritional interventions.

## Introduction

The liver may be affected during the course of many diseases that are systemic or predominantly involve other organs such as rheumatoid arthritis (RA). As an autoinflammatory immune disease, RA is also associated with many extra-articular features of vascular tissue, lung, liver, and other organs (1–3). Clinically, liver lesions have been reported in RA patients, but the cause is unknown (4–6). As the liver is a major metabolic hub, studies addressing the change of metabolism in RA have been partially explored. Liver enzyme elevation has been reported in patients with RA who were not receiving systemic therapy without other diagnoses (7, 8). Hepatic glucokinase activity and glycolysis were increased in arthritic rats, indicating that RA might alter liver glucose metabolism (9). Another study found a decrease in tryptophan and kynurenine levels in the liver of collagen-induced arthritis (CIA) mice (10). Changes in liver lipid metabolism were also observed in rats with adjuvant-induced arthritis (AIA) (11). In the meantime, the immune microenvironment of the liver also was affected by RA. The histology of the liver in RA included Kupffer cell (KC) hyperplasia, mononuclear infiltration around periportal areas, and so on (12). Meanwhile, the function of immune-related proteins synthesized by the liver appears to be compromised. Hepatic albumin synthesis was reduced, resulting in lower serum levels of this protein in RA (13). The opposite was true for C-reactive protein which, like fibrinogen and α2-macroglobulin, was elevated in patients with active RA (14–16). Another study showed that liver hepcidin, which had an immunomodulatory effect, was upregulated during different phases of inflammation in complete Freund’s adjuvant (CFA) rats (17). These hints suggest that the immune function of the liver in RA may be compromised. Although there has been increasing evidence pointing to the changes in the metabolism and immune function of the liver in RA, these studies have only studied the liver in a specific period of RA from a single aspect, and systematic research still remains lacking (3, 18).

Proteomics can investigate and clarify the fundamental laws of life from the perspective of the entire activity of organisms, and it reflects the relationship between molecules and cells, tissues, and the whole biological characteristics (19). In this study, we detected the protein expression in the liver tissue along with the progression of CIA by quantitative proteomics to reveal the function change of the liver in RA development. The comprehensive analyses of hepatic dynamic changes in CIA rats will contribute to the precision therapy for RA.

## Materials and Methods

### Animals

Male Sprague–Dawley (SD) rats (8–10 weeks old) with a mean weight of 180–200 g were purchased from Hua Fukang Biological Polytron Technologies Inc. (Beijing, China) and kept in a specific pathogen-free (SPF) environment (22 ± 1°C, 12 h light/dark cycle). Food and water were made available *ad libitum* under laboratory conditions at 50% ± 10% relative humidity. The animal experiments were carried out following the China-Japan Friendship Hospital’s Animal Care & Welfare Committee protocols (No. zryhyy21-20-12-1).

### Collagen-Induced Arthritis Model Induction

Bovine type II collagen and CFA were purchased from Chondrex (Redmond, WA, USA). The CIA model was established according to previously described methods and protocols (20). In brief, male SD rats were immunized as follows: the primary immunization was given *via* intradermal injection of 200 µl of emulsion composed of equal parts of CFA and 2 mg/ml of bovine type II collagen at the base of the tail on day 0, and the boost immunization consisting of 100 µl of the same emulsion was given on day 7.

The rats were monitored every 2 days, and the arthritis index scores were assigned on a scale of 0 to 4 based on the following criteria (21): 0 = no edema or swelling, 1 = slight edema and erythema limited to the foot and/or ankle, 2 = slight edema and erythema from the ankle to the tarsal bone, 3 = moderate edema and erythema from the ankle to the tarsal bone, and 4 = severe edema and erythema from the ankle to the entire leg. The final score for each rat was the sum of the scores from the two hind limbs, with a maximum possible score of 8. When the rats began to exhibit signs of joint inflammation, the paws with scores of 1–2 were selected as samples for the induction phase. As the disease progressed, the samples for peak were taken from the paws with severe swelling (scores of 3–4). Inflammation in the joints usually subsided after the peak period. The samples for the resolution phase were taken from the paws that had less inflammation (scores of 1–2) (22).

### Histological Analysis

Ankle joints were dissected and immediately fixed in formalin for 3 days, and the left ankle joints were decalcified in 10% EDTA and embedded in paraffin. Hematoxylin and eosin (HE) were used to stain the tissue sections. The histopathological characteristics of the joint were evaluated blindly as described previously (23).

To verify the change in immune function at the peak phase, HE staining was used to study the pathology of liver tissues. Rat liver tissues were collected and fixed in 4% paraformaldehyde and subsequently embedded in paraffin. The sections were stained with HE by using a standard protocol and analyzed under light microscopy.

### Enzyme-Linked Immunosorbent Assay

The levels of TNF-α, IL-1β, and IL-6 in the serum were determined by enzyme-linked immunosorbent assay (ELISA) using commercial kits. The instructions were followed to measure the OD values and calculate the level of expression based on the standard curve. The rat TNF-α, IL-1β, and IL-6 ELISA kits were purchased from Dakewe (Shenzhen, China).

To verify the reliability of the proteomics data, the levels of the lipid metabolism-related differentially expressed proteins (DEPs) with high fold change including ATXN2, TRPM4, and PIK3C2a in the induction phase and the immune function-related DEPs with high fold change such as PGLYRP1, KNG1, and GPNMB at the peak phase in the liver tissue samples of rats were determined by ELISA using commercial kits. The ATXN2, TRPM4, PIK3C2a, PGLYRP1, KNG1, and GPNMB kits were purchased from Fankewei (Shanghai, China).

To verify the change in immune function at the peak phase, the ELISA kits were used to detect the levels of key DEPs of immune function such as C3, MMP-8, S100A9, and CTSZ. The C3, MMP-8, S100A9, and CTSZ kits were purchased from Fankewei (Shanghai, China).

### Proteomics Analysis

The proteomics analysis was performed by Wayen Biotechnologies Inc. (Shanghai, China) according to the following procedure. Frozen liver tissue samples were mechanically homogenized three times for 15 s with a tissue homogenizer before being statically lysed in a protease inhibitor cocktail. The interlayer solution was collected and transferred to a new tube after centrifugation at 13,000 rpm for 20 min at 4°C. To precipitate the solution, six times the volume of 100% acetone was added to the new tube. The solutions were redissolved in a solution of 6 M of guanidine hydrochloride and 300 mM of TEAB. A bicinchoninic acid (BCA) protein assay was used to determine the protein concentration.

The protein samples were then reductively alkylated. The protein solution with 1 M DTT was incubated at 57°C for 1 h before adding 10 µl of 1 M of iodoacetamide/100 µl of solution and storing it at room temperature for 40 min away from light. The alkylated protein was washed three times and digested with trypsin. The peptides were stored at −80°C after digestion. Following centrifugal drying, the peptides were dissolved in 0.1% of acetonitrile and desalted using desalting columns. After vacuum drying, the sample was redissolved in the mobile phase (A phase: 0.1% of formic acid, B phase: 100% of ethyl ester), and the 10–15-µl sample was loaded onto the machine for LC-MS analysis. The separation was performed using an EASY-nLC 1000 chromatograph (Thermo Scientific, USA), with an analytical column (C18, 3 µm, 75 µm × 15 cm) and a flow rate of 300 nl/min. The mass spectrometer was an Orbitrap Fusion Lumos (Thermo Scientific, USA). The data-dependent scanning acquisition mode was used for tandem mass spectrometry detection. The mass-to-charge ratio range is *m*/*z* 350–1,500.

The protein quantitation was performed by the Proteome Discoverer Software 2.4 (Thermo Scientific, USA). We included proteins ≥2 unique peptides in our research to enhance the study’s credibility. These proteins were identified as upregulated or downregulated DEPs when the fold change was ≥1.2 or ≤0.83 and the *p*-value was less than 0.05. The DEP list was then analyzed using online software.

### Ingenuity Pathway Analysis

The ingenuity pathway analysis (IPA, Ingenuity Systems, QIAGEN China Co., Ltd., Shanghai, China; http://www.ingenuity.com) was used to evaluate the trend of protein enrichment changes. The DEPs with fold changes and *p*-values were input into the IPA, and then the function annotations and functional network were algorithmically generated according to protein–protein interactions.

### Targeted Lipid Metabolism Detection

To verify the abnormal lipid metabolism in the induction phase, target metabolomics was used to detect the level of fatty acids (FAs) in the liver. After accurately weighing, approximately 50 mg of liver samples were added to 1 ml of the extract containing the internal standard. TissueLyser was used to grind the samples, and the supernatant was centrifuged at 14,000 rpm for 20 min. The quality control (QC) samples were prepared by combining equal aliquots of one liver sample from each group. The QC sample was examined to ensure that the analysis was stable and repeatable.

All chromatographic separations were carried out using an Agilent ZORBAX SB-C18 (4.6 × 250 mm, 5 µm). The mobile phase consisted of solvent A (10 mM ammonium acetate) and solvent B (10 mM ammonium acetate:70% acetonitrile = 15:85). The total flow rate was 0.2 ml/min, and the following elution gradient was employed: 0–6 min, 25%–40% B; 6–20 min, 40%–70% B; 20–20.1 min, 70%–100% B; 20.1–23 min, 100%–100% B; 23–23.1 min, 100%–25% B; and 23.1–25 min, 25%–25% B. The sample injection volume ranged from 2 to 10 µl. The temperature in the column was maintained at 40°C. The ESI source was used in negative and positive ion modes to generate the MS/MS spectra. The spray voltage was set to 3.5 kV, the ion source temperature to 150°C, and the solvation temperature to 550°C. Scanning was done in the multireaction monitoring (MRM) mode. A total of 18 FAs were measured, namely, C12:0, C12:1, C14:0, C14:1, C15:0, C16:0, C16:1, C17:0, C18:0, C18:1, C18:2, C18:3, C20:0, C20:4, C20:5, C22:0, C22:2, and C22:6. The detailed mass spectrometric parameters of FAs are provided in **Supplementary Table 1**.

### Statistical Analysis

GraphPad Prism 8 was used to analyze the data. All data were expressed as mean ± SD. For the statistical analysis, the *t*-test or one-way analysis of variance was used. Statistical significance was defined as *p <*0.05.

## Results

### Dynamic Change of Arthritis During the Progression of CIA

**Figure 1:**
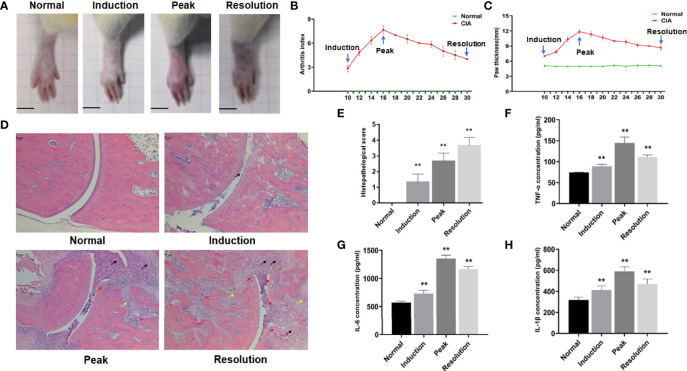
The dynamic changes of arthritis in collagen-induced arthritis (CIA) rats. **(A)** Photographs of the hind paw and ankle joint of rats. **(B)** Arthritis score. **(C)** Hind paw thickness, scale bar = 1 cm. **(D)** The representative histopathological finding of the ankle joint from each group (×50), scale bar = 50 μm. The black arrow indicated inflammatory cell infiltration, the blue arrow indicated synovial hyperplasia, the green arrow indicated pannus formation, the yellow arrow indicated bone destruction, and the red arrow indicated cartilage erosion. **(E)** Histopathological score. (**F–H**) The levels of TNF-α, IL-6, and IL-1β in the serum of rats from each group. Data were mean ± SD. *N* = 6. **p < 0.01 compared with the normal group.

Following boost immunization, the arthritis index (AI) and hind paw thickness were detected to assess joint swelling in the CIA models. In collagen-treated rats, arthritis appeared initially in the induction phase. As the disease advanced in CIA rats, the symptoms of arthritis became more severe, with the AI and hind paw thickness approaching their maximum. Following this, the symptoms of arthritis gradually alleviated in the resolution phase of CIA, but it remained more severe than in the induction phase (**Figure 1A**), and the AI, as well as the hind paw thickness, showed the same trend (**Figures 1B, C**).

The dynamic change of arthritis was also visible in the histopathological sections of ankle joints from CIA rats. The results revealed only a minor amount of inflammatory cell infiltration and synovial hyperplasia in the ankle joint of CIA rats after induction. As arthritis symptoms worsened, inflammatory cell infiltration, pannus formation, cartilage erosion, and bone destruction were observed in the ankle joint of CIA rats at the peak phase. When the disease progressed to the resolution phase, these pathological features of arthritis were further aggravated in CIA rats (**Figure 1D**), and the histopathological score reflected this trend (**Figure 1E**).

ELISA was also used to detect inflammatory factors in the serum of CIA rats. The levels of serum inflammatory factors such as TNF-α, IL-6, and IL-1β partially reflected the inflammatory response during disease procession. The results showed that the levels of TNF-α, IL-6, and IL-1β in CIA rats were significantly upregulated when compared with the normal group (**Figures 1F–H**).

### Identification of DEPs in the Liver During Arthritis Development

**Figure 2:**
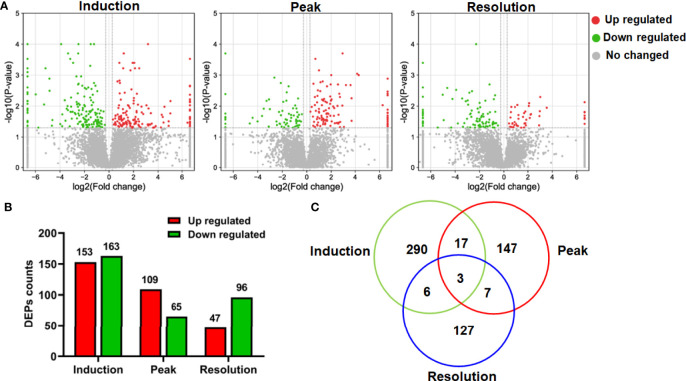
The differentially expressed proteins (DEPs) in the proteomics data. **(A)** The volcano plot depicted the DEPs at different phases. **(B)** The number of upregulated and downregulated DEPs at different phases. **(C)** The Venn diagram showed protein identification overlap at different phases.

Having examined the symptoms of arthritis as the disease progressed, we aimed to systematically describe the changes in hepatic function using global protein expression profiling; the corresponding volcano plot is shown in **Figure 2A**. When compared to the normal group, 316, 174, and 143 proteins were identified as DEPs at the three stages of CIA, respectively (**Figure 2B**). The findings also revealed that the counts of DEPs in the liver decreased as arthritis progressed. Interestingly, only a small number of DEPs were found to be standard across the three phases of CIA using the Venn diagram, implying that the differentially regulated proteins may be stage-specific (**Figure 2C**). As a result, we used bioinformatics analysis to investigate the DEPs further.

### Identification of Function Annotations Showing the Significant Enrichment of DEPs in the Liver

**Figure 3:**
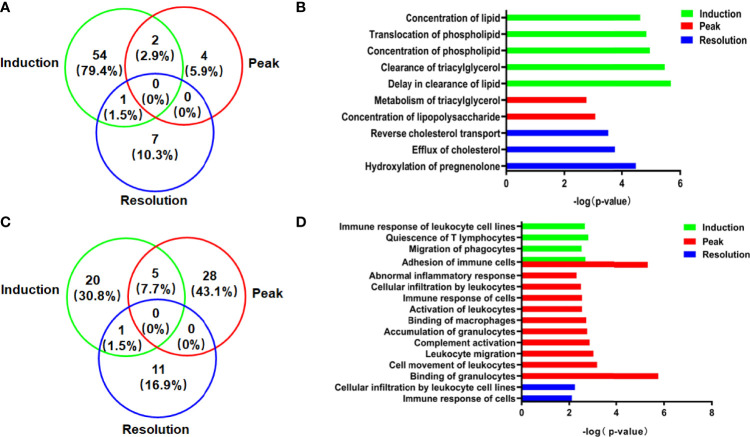
The DEPs and function annotations related to lipid metabolism or immune function in the model group vs. the normal group in different phases. **(A)** The Venn diagrams showed the number and proportion of DEPs related to lipid metabolism. **(B)** The function annotations related to lipid metabolism showing enrichment of DEPs. **(C)** The Venn diagrams showed the number and proportion of DEP related to immune functions. **(D)** The function annotations related to immune function showing enrichment of DEPs.

The DEPs were input into the IPA software, and the function enrichment [−log(*p*-value, 10) > 1.3] lists were output to assess the essential function of DEPs in liver tissue. Because metabolism and immune function are closely linked to the liver, the function enrichment annotations associated with them were given special attention in this study. As demonstrated in **Figure 3B**, lipid metabolism in the liver was affected in different phases of CIA rats. In the induction phase, there were 35 function enrichment annotations of lipid metabolism (**Supplementary Table 2**). The top 5 function enrichment annotations were “delay in the clearance of lipid,” “clearance of triacylglycerol,” “concentration of a phospholipid,” “translocation of a phospholipid,” and “concentration of lipid” (**Figure 3B**). Meanwhile, compared to the other phases, the number of DEPs and function annotations associated with lipid metabolism in the induction phase was the highest (**Figure 3A**). At the peak phase, the lipid metabolism enrichment annotations were “concentration of lipopolysaccharide” and “metabolism of triacylglycerol.” The lipid metabolism function enrichment annotations in the resolution phase were “hydroxylation of pregnenolone,” “efflux of cholesterol,” and “reverse cholesterol transport” (**Figure 3B**).

In addition to lipid metabolism, the immune function was affected in the CIA model, particularly at the peak phase of CIA (**Figure 3C**). The immune functions associated with DEPs in the induction enrichment were “quiescence of T lymphocytes,” “adhesion of immune cells,” “migration of phagocytes,” and “immune response of leukocyte cell lines.” The effect on liver immune function of CIA rats was particularly significant at the peak phase. The function enrichment annotations of immune function were “binding of granulocytes,” “adhesion of immune cells,” “cell movement of leukocytes,” “leukocyte migration,” “complement activation,” “accumulation of granulocytes,” “binding of macrophages,” “activation of leukocytes,” “immune response of cells,” “cellular infiltration by leukocytes,” and “abnormal inflammatory response,” while the immune functions associated with the enrichment of DEPs were “cellular infiltration by leukocyte cell lines” and “immune response of cells” in the resolution phase (**Figure 3D**).

### Changes in Lipid Metabolic Function in the Induction Phase of CIA

**Figure 4:**
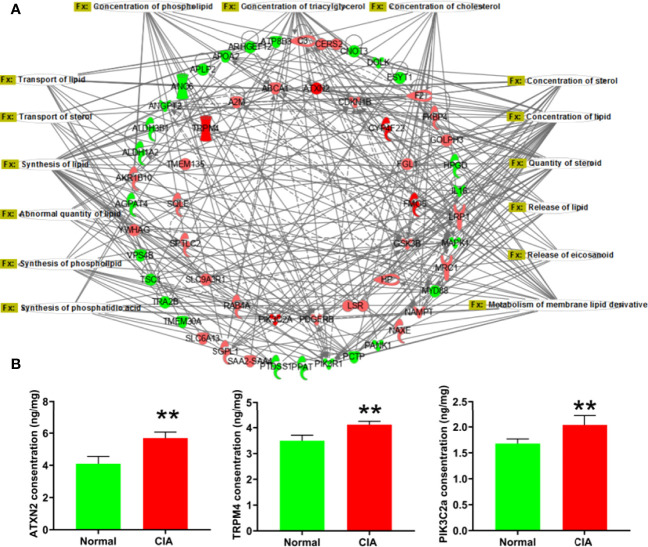
The lipid metabolism network and the verification of representative DEPs in the induction phase. **(A)** The lipid metabolism network: the red color represented the upregulation of protein expression in the model group vs. the normal group and the green color represented the downregulation of protein expression in the model group vs. the normal group. **(B)** The verification of DEPs related to lipid metabolism. Data were expressed as mean ± SD. *N* = 6. **p < 0.01 compared with the normal group.

According to the IPA analysis findings, the DEPs were closely related to the lipid metabolic function during the different phases of arthritis development in CIA rats. In the induction phase, most of the DEPs and the −log(*p*-values) of lipid metabolic function annotations demonstrated that the lipid metabolic function of the liver had changed significantly. To further explore the relationship between the DEPs and lipid metabolic function, the lipid metabolism network was exported from the IPA (**Figure 4A**). To verify the reliability of proteomics, we firstly experimentally identified DEPs with a high fold change related to lipid metabolism such as ATXN2, TRPM4, and PIK3C2a. **Figure 4B** displays that the levels of ATXN2, TRPM4, and PIK3C2a were all obviously elevated in the liver of CIA rats. The changes in the expression levels of these proteins were consistent with the tendencies observed in the proteomics analysis (**Supplementary Table 3**).

**Figure 5:**
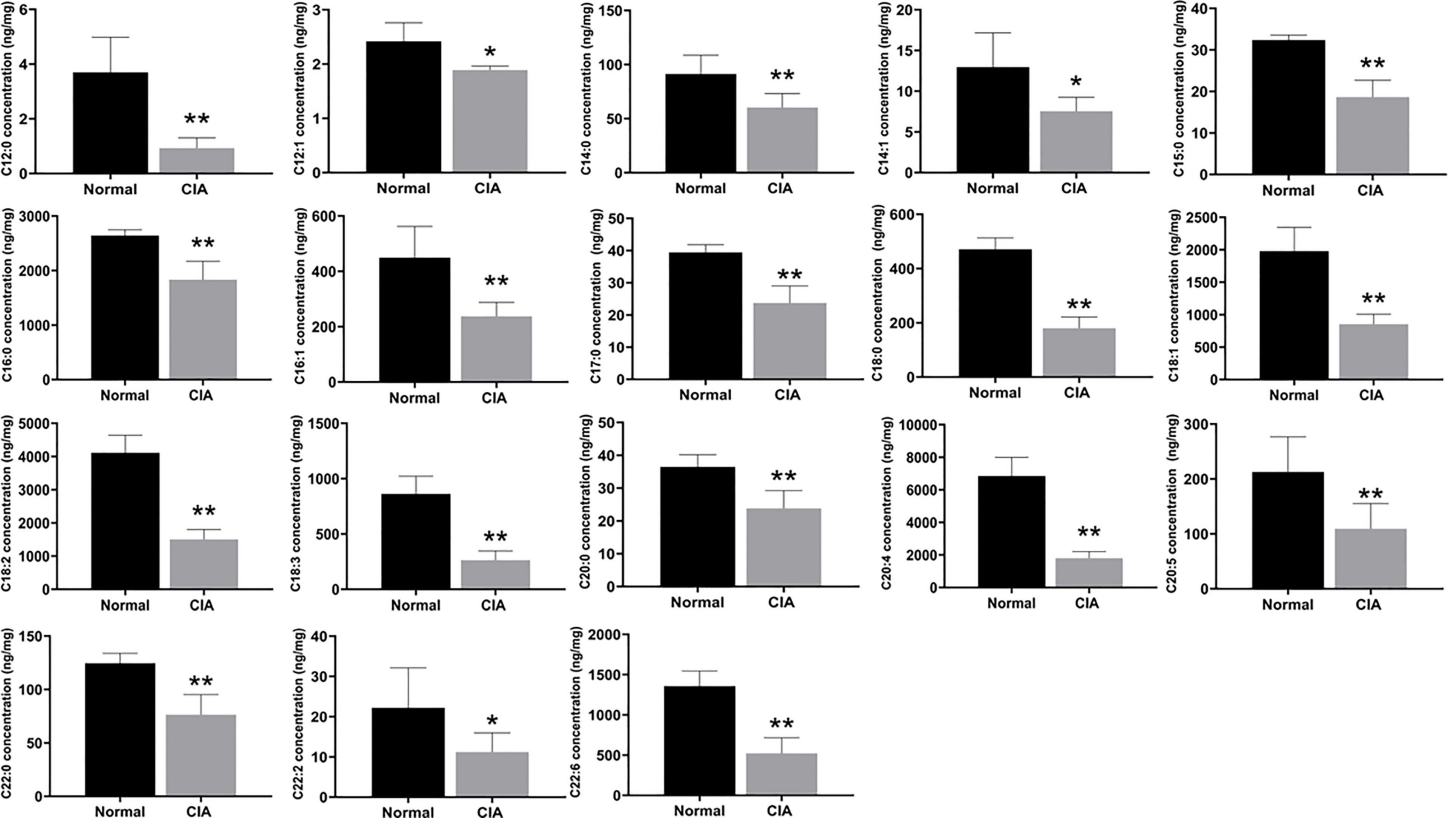
The levels of FAs in the liver of CIA rats in the induction phase. Data were expressed as mean ± SD. *N* = 6. *p < 0.05, **p < 0.01 compared with the normal group.

Then, we used targeted FAs analysis to confirm the levels of FAs in order to further explain the effect of lipid metabolism of the liver in the induction phase. The 18 FAs (C12:0, C12:1, C14:0, C14:1, C15:0, C16:0, C16:1, C17:0, C18:0, C18:1, C18:2, C18:3, C20:0, C20:4, C20:5, C22:0, C22:2, and C22:6), the majority of which contained more than 12 carbons, were decreased significantly in the liver of CIA rats in the induction phase (**Figure 5**). Therefore, the results indicated that the levels of most FAs were altered during the induction phase of CIA rats.

### Changes in Immune Function at the Peak Phase of CIA

**Figure 6:**
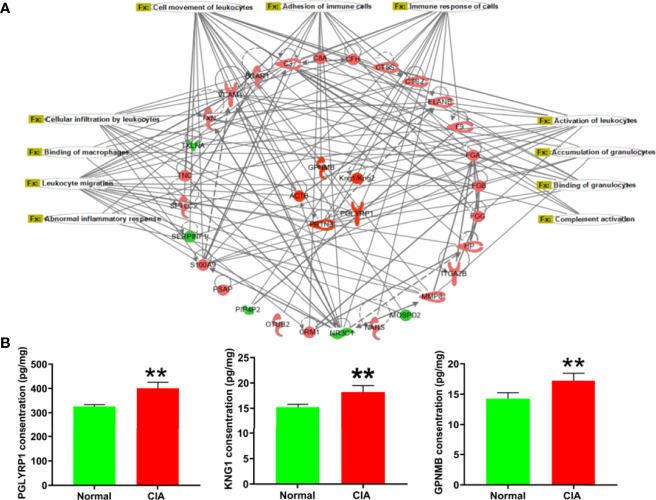
The immune-related function network and the verification of representative DEPs in the peak phase. **(A)** The immune-related function network: the red color represented the upregulation of protein expression in the model group vs. the normal group and the green color represented the downregulation of protein expression in the model group vs. the normal group. **(B)** The verification of DEPs related to immune function. Data were mean ± SD. *N* = 6. **p < 0.01 compared with the normal group.

According to the IPA analysis findings, the DEPs were closely related to the immune function during the different phases of arthritis development in CIA rats. At the peak phase, most of the DEPs and the −log(*p*-values) of immune-related function annotations demonstrated that the immune function of the liver had changed significantly. To further explore the relationship between the DEPs and immune function, the immune network was exported from the IPA (**Figure 6A**). First, we used ELISA kits to confirm the expression of DEPs with high fold change in the immune function network. The DEPs, including PGLYRP1, KNG1, and GPNMB, were remarkably elevated in the livers of CIA rats (**Figure 6B**). The changes in the expression levels of these proteins were consistent with the tendencies observed in the proteomics analysis. The fold changes and *p*-values of these DEPs are listed in **Supplementary Table 4**.

**Figure 7:**
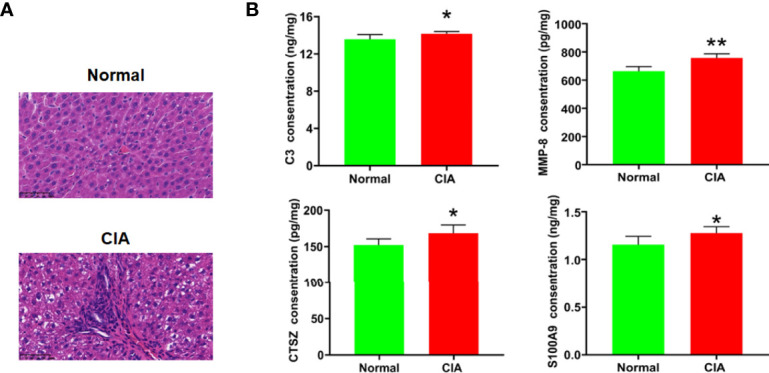
Verification of the change of immune function in the liver of CIA rats in the peak phase. **(A)** HE staining of the liver (×400), scale bar = 50 μm. **(B)** The verification of key DEPs related to complement activation and migration or adhesion of immune cells. Data were expressed as mean ± SD. *N* = 6. *p < 0.05, **p < 0.01 compared with the normal group.

As the functional enrichment analysis showed that immune cell infiltration and inflammatory response were influenced at the peak phase, HE staining was used for further confirmation. The result showed that a large number of immune cells infiltrating the liver tissue could be seen in the model group at the peak phase (**Figure 7A**). In addition, complement activation and migration or adhesion of immune cells were also affected at the peak phase in the functional enrichment analysis. We therefore used ELISA to verify these results. We found that C3, the key DEP for complement activation, was increased significantly in the CIA model group when compared with the normal group. The key DEPs related to migration or adhesion of immune cells such as MMP-8, CTSZ, and S100A9 were also increased significantly in the CIA model group when compared with the normal group (**Figure 7B**).

## Discussion

The CIA model is recognized as the classical animal model to study human RA, which has great similarities with human RA in pathology, immunology, and clinical features (24). The wide application of this model provides more help in exploring the pathogenesis and pathological process of RA and the treatment plan for RA. This study demonstrated that the dynamics of arthritis symptoms in CIA, including the AI, hind paw thickness, joint swelling, inflammation, and histopathology, occurred during the induction phase and persisted if not treated. The dynamic outcome was essentially consistent with previous research on the CIA model (25, 26). This demonstrated that CIA is an ideal model to investigate the pathological and mechanism changes during the development of RA, primarily during the following three phases: induction, peak, and resolution (27).

The liver is the body’s most extensive solid organ essential for metabolism and immune function (28, 29). Clinically, liver lesions have been reported in RA patients, but the cause is unknown (4–6). Previous studies showed that oxidative stress, differences in metabolic enzyme expression, gluconeogenesis, and other factors exist in the livers of animal models associated with RA (30, 31). Because the condition of RA changes dynamically, studying only one phase is insufficient. Therefore, in this study, dynamic proteomics analysis was used to gain a complete understanding of the liver condition during the progression of RA. The results revealed that lipid metabolism in the liver was significantly influenced, particularly in the induction phase of CIA, and mainly involved the metabolism of cholesterol, phospholipid, triglyceride (TG), and so on. RA is a chronic systemic autoimmune disease in which approximately 60% of patients have dyslipidemia (32–35). Previous research showed that low-density lipoprotein (LDL), very-low-density lipoprotein (VLDL), and unsaturated lipid levels in the urine of CIA rats were significantly lower, implying that changes in lipid metabolism occurred in RA (36). Clinical studies have revealed elevated levels of FAs and cholesterol in RA patients’ sera (37). The reasons for this could be multifaceted, referring to multiple lipid metabolism-related organs, particularly the liver, which is at the center of regulating lipid metabolism (38, 39). A study showed that dysregulated lipid metabolism of the liver existed in AIA rats (40). However, the precise molecular mechanism of these influences is unknown and requires further investigation. Our dynamic proteomic discovery added a small amount of content to this research in this area of investigation. Through further analysis of the lipid metabolism network, we found some key DEPs related to lipid metabolism, including PIK3C2α, TRPM4, ATXN2, and so on. PIK3C2a is a gene that encodes a lipid kinase involved in regulating fat production, and increasing its protein levels may affect FA synthesis (41). TRPM4 is primarily expressed in adipose tissue-derived stem cells, and its downregulation alters histamine-induced intracellular calcium concentration, implying that it is involved in adipogenesis (42, 43). ATXN2 is a pleiotropic gene linked to various diseases, including diabetes and inflammation (44). A recent study found that overexpression of circ-ATXN2 promoted adipogenesis in adipose tissue-derived stromal cells (45). When its activity increased, ATP Binding Cassette Subfamily A Member 1 (ABCA1), a key transporter of cholesterol out of the cell, triggered the metabolism of cholesterol and phospholipids in cells and influenced the process of FA differentiation (46, 47). These DEPs acted in concert, causing an imbalance in lipid synthesis and catabolism in the livers of CIA rats.

Because the effects on lipid metabolism in the liver of CIA rats were complex and the experimental conditions were limited, only FA levels in the liver were further detected to explain the change of lipid metabolism in RA in our study. FAs play a variety of roles in biological processes, particularly in autoimmune diseases such as RA (48). For instance, n−3 FAs, primarily eicosapentaenoic acid (EPA) and docosahexaenoic acid (DHA), improved arthritis symptoms in RA patients by inhibiting inflammation and immunomodulation, while palmitic acid (PA) influenced the balance of bone metabolism from autophagy and apoptosis (49, 50). Therefore, understanding the levels of hepatic FAs provided a more theoretical basis to explore the changes in lipid metabolism in RA. Targeted metabolomics results showed that most FAs in the liver of CIA rats in the induction phase were at a lower level which revealed the imbalance of FA metabolism. The relative stability of FAs necessitates a dynamic balance between fat synthesis and catabolism, which is influenced by various factors (51). The complex biological process of FAs in the liver consists mainly of synthesis and decomposition. The intake of free FAs from food and adipose tissue *via* the liver is one of the two main sources of FAs; the other is *de-novo* lipogenesis. Meanwhile, oxidative decomposition is used to prevent excessive FA accumulation in the liver. Inflammation has been shown in studies to accelerate the decomposition of peripheral adipose tissue and increase FAs, which may be responsible for the elevated levels of FAs in RA serum (52, 53). Although free FAs in the peripheral tissues increased, most of these in the liver decreased in CIA rats. One possible explanation is that the carrier for FAs entering the liver has changed. Previous research found that mRNA expression of the constitutive androstane receptor decreased in the liver of mice with CIA, even though it is an important carrier for FAs to enter the mitochondria (54). Aside from that, the NADPH/NADP^+^ ratio may also influence FA synthesis. Meanwhile, the ratio was reduced in fed and fasted rats with AIA when the liver was subjected to oxidative stress (55–57). On the other hand, liver peroxidation aided in the breakdown of FAs. A series of events culminated in the reduction of FAs in arthritis models. The imbalance between FA synthesis and breakdown may be the primary cause of the decrease of FAs in the liver of arthritis models, and the conversion of amino acids, glucose, and other substances may be involved. This was largely consistent with the findings of the study of Wendt et al., which showed that hepatic lipid metabolism in rats with AIA was shifted toward a catabolic state instead of other chronic wasting diseases (11).

Apart from the imbalance of lipid metabolism in the liver, pathogenic substances such as cytokines and interferon produced during RA may also impact the liver immune function (58). Our proteomics results revealed that the liver immune function of arthritic rats had changed, particularly at the CIA peak phase. In our study, the key immune-related DEPs that have been verified were PGLYRP1, KNG1, and GPNMB. PGLYRP1 is a protein that activates innate immunity and is stored in neutrophil gelatinase granules, which plays an essential role in phagocytosis and sterilization (59). Meanwhile, Luo et al. found that PGLYRP1 in RA patients’ sera was correlated with rheumatoid factor and anti-cyclic citrullinated peptide, and the increased expression could serve as a potential biomarker for RA diagnosis (60). However, it is unknown whether the increase of PGLYRP1 in liver tissue was associated with blood in CIA rats. KNG1 is a macromolecular inflammatory response mediator produced primarily by liver cells (61). A previous study found that KNG1 overexpression exacerbated oxidative stress and mitochondrial damage (62). Singh et al. (63) predicted that KNG1 may be the potential target of RA by bioinformatics, which requires further experimental verification. The level of GPNMB is low in normal hepatocytes but higher in hepatocellular carcinoma cells according to research (64, 65). GPNMB is involved in immune system function and is highly expressed in a variety of innate immunity cells, which may play an important role in the pathogenesis of autoimmune diseases (66).

The analysis of IPA showed that the functions of DEPs related to immune function mainly included “binding of granulocytes,” “adhesion of immune cells,” “cell movement of leukocytes,” “leukocyte migration,” “complement activation,” “accumulation of granulocytes,” “binding of macrophages,” “activation of leukocytes,” “immune response of cells,” “cellular infiltration by leukocytes,” and “abnormal inflammatory response.” This demonstrated that immunologic insults did not exist solely in the circulating blood and joints of RA (67, 68). A few animal studies have demonstrated that immune cells in the liver of arthritis models changed. To be more specific, iNKT cells were only activated at the early phase of the onset of arthritis in mice, while they did not change in the spleen and lymph nodes (69). Compared to mice with arthritis in the control group, the lack of KCs reduced arthritis score and joint swelling but maintained joint space improvement (70). Our proteomics studies showed that liver immune functions such as cellular infiltration, inflammatory response, complement activation, and migration and adhesion of immune cells were affected in the CIA rats at the peak phase. The HE staining results confirmed the accumulation and infiltration of immune-related cells in the liver of CIA rats compared with the normal group. This was consistent with the proteomic results of the infiltration of immune cells and inflammatory response. In addition, we found that the levels of C3, MMP-8, CTSZ, and S100A9 were significantly elevated in the liver of CIA rats. C3 is a core hub of the complement activation pathway. A clinical study showed that the serum level of complement component C3 was increased in patients with active untreated RA (71). C3 can be produced by hepatocytes, macrophages, and so on and has been proven to play an important role in multiple immunological processes including accumulation of immune complexes, aggregation of immune cells, inflammation, and so on (72). MMP-8 is also known as collagenase-2 or neutrophil collagenase, which can be produced by neutrophils (73). Research has shown that the level of MMP-8 in synovial fluid was low in the early phase and increased with the progression of RA (74). Previous studies have demonstrated that the increase of MMP-8 activity enhanced synovial fibroblast migration across collagen type I in RA (75). CTSZ was predominantly expressed by monocytes, macrophages, and dendritic cells, and it could enhance the adhesion of monocytes/macrophages to fibrinogen as well as the maturation of dendritic cells, a process crucial in the initiation of adaptive immunity (76). Studies found that CTSZ was specifically increased in the synovial membrane of RA and involved in modulating the attachment of migrating cells to the ECM component (77, 78). S100A9 is a Ca(2+)-binding protein of innate immune response and is tightly associated with pro-inflammatory and adhesion functions of neutrophils (79). It has been found at high concentrations in the synovial fluid of patients with RA which is known as damage-associated molecular patterns (80). These molecules interact with each other to influence the hepatic immune function of CIA rats in the peak phase. Abnormalities of immune cells in RA disease may be related to the DEPs that were detected by proteomics, and this will be the focus of our next research.

Our studies provided additional evidence for the changes in the immune function of CIA rats, particularly when the disease was at its peak. There may exist a close connection between the change of immune function and immune cells in the liver of the RA model and further research is needed. Although the quantitative proteomics revealed the dynamic changes in hepatic lipid metabolism and immune function during the development of CIA, we only validated the dysregulated lipid metabolism in the induction phase and the immune function at the peak phase. The dynamic changes of lipid metabolism and immune function will be further studied in our future work.

## Conclusion

In conclusion, this study revealed that lipid metabolism of the liver was affected, particularly in the induction phase of CIA, and that an obvious change of immune function existed in the liver of CIA rats, particularly at the peak phase. Such a condition should be considered in therapeutic interventions, especially the use of drugs that may affect the liver.

## Data Availability Statement

The original contributions presented in the study are publicly available. The data can be found here: http://proteomecentral.proteomexchange.org/cgi/GetDataset?ID=PXD032872.

## Ethics Statement

The animal study was reviewed and approved by the China-Japan Friendship Hospital’s Animal Care & Welfare Committee.

## Author Contributions

YS and JS performed the major research. ZN performed the targeted lipid metabolism detection. DF, HS, and HZ collected the samples. LL, NZ, and CL provided technical support. AL and XH designed the study and revised the manuscript. All authors contributed to the article and approved the submitted version.

## Funding

This research was supported by the Fundamental Research Funds for the Central Public Welfare Research Institutes (Z0736) and the National Key R&D Program of China (2018YFC1705205).

## Conflict of Interest

The authors declare that the research was conducted in the absence of any commercial or financial relationships that could be construed as a potential conflict of interest.

## Publisher’s Note

All claims expressed in this article are solely those of the authors and do not necessarily represent those of their affiliated organizations, or those of the publisher, the editors and the reviewers. Any product that may be evaluated in this article, or claim that may be made by its manufacturer, is not guaranteed or endorsed by the publisher.

## References

[1] Zou K Xiao FK Li HY Zhou Q Ban L Yang M. 'Risk of Cardiovascular Disease in Chinese Patients With Rheumatoid Arthritis: A Cross-Sectional Study Based on Hospital Medical Records in 10 Years'. PLoS One (2017) 12:e0180376. doi: 10.1371/journal.pone.0180376 28678807 PMC5498026

[2] Zheng B Soares de Moura C Machado M Pineau CA Curtis JR Vinet E. 'Association Between Chronic Obstructive Pulmonary Disease, Smoking, and Interstitial Lung Disease Onset in Rheumatoid Arthritis'. Clin Exp Rheumatol (2021). doi: 10.55563/clinexprheumatol/i9au1r 34494959

[3] Comar JF Babeto de Sá-Nakanishi A de Oliveira AL Marques Nogueira Wendt M Bersani Amado CA Ishii Iwamoto EL. 'Oxidative State of the Liver of Rats With Adjuvant-Induced Arthritis'. Free Radic Biol Med (2013) 58:144–53. doi: 10.1016/j.freeradbiomed.2012.12.003 23246655

[4] Whaley K Goudie RB Williamson J Nuki G Dick WC Buchanan WW. 'Liver Disease in Sjögren's Syndrome and Rheumatoid Arthritis'. Lancet (1970) 1:861–3. doi: 10.1016/S0140-6736(70)91690-9 4191507

[5] Watanabe R Igarashi T Takahashi T Kondo H Okazaki S Kudo M. 'Multidisciplinary Approach to Prevent *De Novo* Hepatitis B in Patients With Rheumatoid Arthritis'. Tohoku J Exp Med (2020) 252:133–41. doi: 10.1620/tjem.252.133 33028756

[6] Tiger LH Gordon MH Ehrlich GE Shapiro B. 'Liver Enlargement Demonstrated by Scintigraphy in Rheumatoid Arthritis'. J Rheumatol (1976) 3:15–20. 818377

[7] Fernandes L Sullivan S McFarlane IG Wojcicka BM Warnes TW Eddleston AL. 'Studies on the Frequency and Pathogenesis of Liver Involvement in Rheumatoid Arthritis'. Ann Rheum Dis (1979) 38:501–6. doi: 10.1136/ard.38.6.501 PMC1000409 539842

[8] Kendall MJ Cockel R Becker J Hawkins CF Annals of the Rheumatic Diseases Hawkins. 'Raised Serum Alkaline Phosphatase in Rheumatoid Disease. An Index of Liver Dysfunction?'. Ann Rheum Dis (1970) 29:537. doi: 10.1136/ard.29.5.537 5476681 PMC1010569

[9] Sá-Nakanishi AB Soni-Neto J Moreira LS Gonçalves GA Silva FMS Bracht L. 'Anti-Inflammatory and Antioxidant Actions of Methyl Jasmonate Are Associated With Metabolic Modifications in the Liver of Arthritic Rats'. Oxid Med Cell Longev (2018) 2018:2056250. doi: 10.1155/2018/2056250 30210649 PMC6126068

[10] Kolodziej L. 'Systemic Metabolism of Tryptophan and its Catabolites, Kynurenine and 3-HAA, in Mice With Inflammatory Arthritis'. Gene (2013) 512:23–7. doi: 10.1016/j.gene.2012.09.122 23063938

[11] Wendt MMN de Oliveira MC Franco-Salla GB Castro LS Parizotto Â V Souza Silva FM. 'Fatty Acids Uptake and Oxidation are Increased in the Liver of Rats With Adjuvant-Induced Arthritis'. Biochim Biophys Acta Mol Basis Dis (2019) 1865:696–707. doi: 10.1016/j.bbadis.2018.12.019 30593897

[12] Lefkovits AM Farrow IJ. 'The Liver in Rheumatoid Arthritis'. Ann Rheum Dis (1955) 14:162–9. doi: 10.1136/ard.14.2.162 PMC1006794 14388592

[13] Tigka E Daskala I Rallis G Anagnostopoulou S Tesseromatis C. 'Adjuvant Arthritis-Induced Changes on Ampicillin Binding in Serum and Tissues Under the Influence of non-Steroidal Anti-Inflammatory Drugs in Rats'. Eur J Drug Metab Pharmacokinet (2005) 30:235–41. doi: 10.1007/BF03190626 16435567

[14] Fang Z Lv J Wang J Qin Q He J Wang M. 'C-Reactive Protein Promotes the Activation of Fibroblast-Like Synoviocytes From Patients With Rheumatoid Arthritis'. Front Immunol (2020) 11:958. doi: 10.3389/fimmu.2020.00958 32508836 PMC7251027

[15] Jin T Bokarewa M Amu S Tarkowski A. 'Impact of Short-Term Therapies With Biologics on Prothrombotic Biomarkers in Rheumatoid Arthritis'. Clin Exp Rheumatol (2009) 27:491–4. 19604443

[16] Zhao KW Murray EJ Murray SS. 'Fibroblastic Synoviocytes Secrete Plasma Proteins *via* α2 -Macroglobulins Serving as Intracellular and Extracellular Chaperones'. J Cell Biochem (2015) 116:2563–76. doi: 10.1002/jcb.25201 25900303

[17] Nazemian V Kalanaky S Manaheji H Houshmandi E Mohammadi M Zaringhalam J. 'Anti-Hyperalgesia Effect of Nanchelating Based Nano Particle, RAc1, can be Mediated *via* Liver Hepcidin Expression Modulation During Persistent Inflammation'. Int Immunopharmacol (2019) 69:337–46. doi: 10.1016/j.intimp.2019.02.003 30776642

[18] Song YL Foster WR Shuster DJ Nadler SG Salter-Cid L Sasseville VG. 'Transcriptional Profiling of Liver and Effect of Glucocorticoids in a Rat Adjuvant-Induced Arthritis Model'. Vet Pathol (2011) 48:885–95. doi: 10.1177/0300985810390018 21149847

[19] Zürbig P Jahn H. 'Use of Proteomic Methods in the Analysis of Human Body Fluids in Alzheimer Research'. Electrophoresis (2012) 33:3617–30. doi: 10.1002/elps.201200360 23160951

[20] Holmdahl R Rubin K Klareskog L Dencker L Gustafson G Larsson E. 'Appearance of Different Lymphoid Cells in Synovial Tissue and in Peripheral Blood During the Course of Collagen II-Induced Arthritis in Rats'. Scand J Immunol (1985) 21:197–204. doi: 10.1111/j.1365-3083.1985.tb01421.x 3158067

[21] Holmdahl R Jansson L Andersson M Larsson E. 'Immunogenetics of Type II Collagen Autoimmunity and Susceptibility to Collagen Arthritis'. Immunology (1988) 65:305–10. PMC1384929 3192274

[22] Booth G Newham P Barlow R Raines S Zheng B Han S. 'Gene Expression Profiles at Different Stages of Collagen-Induced Arthritis'. Autoimmunity (2008) 41:512–21. doi: 10.1080/08916930802095210 18608173

[23] Deng Y Luo H Shu J Shu H Lu C Zhao N. 'Pien Tze Huang Alleviate the Joint Inflammation in Collagen-Induced Arthritis Mice'. Chin Med (2020) 15:30. doi: 10.1186/s13020-020-00311-3 32256686 PMC7106633

[24] Zhang Q Peng W Wei S Wei D Li R Liu J. 'Guizhi-Shaoyao-Zhimu Decoction Possesses Anti-Arthritic Effects on Type II Collagen-Induced Arthritis in Rats *via* Suppression of Inflammatory Reactions, Inhibition of Invasion & Migration and Induction of Apoptosis in Synovial Fibroblasts'. BioMed Pharmacother (2019) 118:109367. doi: 10.1016/j.biopha.2019.109367 31545276

[25] Wang T Li J Jin Z Wu F Li Y Wang X. 'Dynamic Frequency of Blood CD4+CD25+ Regulatory T Cells in Rats With Collagen-Induced Arthritis'. Korean J Physiol Pharmacol (2015) 19:83–8. doi: 10.4196/kjpp.2015.19.2.83 PMC4342740 25729268

[26] Chu Y Wang J Zhou X. 'Mast Cell Chymase in Synovial Fluid of Collagen-Induced-Arthritis Rats Regulates Gelatinase Release and Promotes Synovial Fibroblasts Proliferation *via* FAK/p21 Signaling Pathway'. Biochem Biophys Res Commun (2019) 514:336–43. doi: 10.1016/j.bbrc.2019.04.121 31036322

[27] Zhou Z Miao Z Luo A Zhu D Lu Y Li P. 'Identifying a Marked Inflammation Mediated Cardiac Dysfunction During the Development of Arthritis in Collagen-Induced Arthritis Mice'. Clin Exp Rheumatol (2020) 38:203–11. 31140393

[28] Wong YC Tay SS McCaughan GW Bowen DG Bertolino P. 'Immune Outcomes in the Liver: Is CD8 T Cell Fate Determined by the Environment?'. J Hepatol (2015) 63:1005–14. doi: 10.1016/j.jhep.2015.05.033 26103545

[29] Zheng M Tian Z. 'Liver-Mediated Adaptive Immune Tolerance'. Front Immunol (2019) 10:2525. doi: 10.3389/fimmu.2019.02525 31787967 PMC6856635

[30] Toda A Kihara T Ono N Nagamatsu A Shimeno H. 'Liver Haem Metabolism in Adjuvant-Induced Arthritic Rats'. Xenobiotica (1996) 26:415–23. doi: 10.3109/00498259609046720 9173682

[31] Lin CH Hsu KW Chen CH Uang YS Lin CJ. 'Differential Changes in the Pharmacokinetics of Statins in Collagen-Induced Arthritis Rats'. Biochem Pharmacol (2017) 142:216–28. doi: 10.1016/j.bcp.2017.06.118 28636885

[32] Lauper K Gabay C. 'Cardiovascular Risk in Patients With Rheumatoid Arthritis'. Semin Immunopathol (2017) 39:447–59. doi: 10.1007/s00281-017-0632-2 28455580

[33] Lanchais K Capel F Tournadre A. 'Could Omega 3 Fatty Acids Preserve Muscle Health in Rheumatoid Arthritis?'. Nutrients (2020) 12(1):223. doi: 10.3390/nu12010223 PMC7019846 31952247

[34] Kim D Chung H Lee JE Kim J Hwang J Chung Y. 'Immunologic Aspects of Dyslipidemia: A Critical Regulator of Adaptive Immunity and Immune Disorders'. J Lipid Atheroscler (2021) 10:184–201. doi: 10.12997/jla.2021.10.2.184 34095011 PMC8159760

[35] Roubenoff R Roubenoff RA Cannon JG Kehayias JJ Zhuang H Dawson-Hughes B. 'Rheumatoid Cachexia: Cytokine-Driven Hypermetabolism Accompanying Reduced Body Cell Mass in Chronic Inflammation'. J Clin Invest (1994) 93:2379–86. doi: 10.1172/JCI117244 PMC294444 8200971

[36] Yun X Dong S Hu Q Dai Y Xia Y. '(1)H NMR-Based Metabolomics Approach to Investigate the Urine Samples of Collagen-Induced Arthritis Rats and the Intervention of Tetrandrine'. J Pharm BioMed Anal (2018) 154:302–11. doi: 10.1016/j.jpba.2018.03.026 29567573

[37] Zhou J Chen J Hu C Xie Z Li H Wei S. 'Exploration of the Serum Metabolite Signature in Patients With Rheumatoid Arthritis Using Gas Chromatography-Mass Spectrometry'. J Pharm BioMed Anal (2016) 127:60–7. doi: 10.1016/j.jpba.2016.02.004 26879423

[38] Zeng Y Ren K Zhu X Zheng Z Yi G. 'Long Noncoding RNAs: Advances in Lipid Metabolism'. Adv Clin Chem (2018) 87:1–36. doi: 10.1016/bs.acc.2018.07.001 30342708

[39] You M Arteel GE. 'Effect of Ethanol on Lipid Metabolism'. J Hepatol (2019) 70:237–48. doi: 10.1016/j.jhep.2018.10.037 PMC6436537 30658725

[40] Xie Y Feng SL Mai CT Zheng YF Wang H Liu ZQ. 'Suppression of Up-Regulated Lxrα by Silybin Ameliorates Experimental Rheumatoid Arthritis and Abnormal Lipid Metabolism'. Phytomedicine (2021) 80:153339. doi: 10.1016/j.phymed.2020.153339 33038868

[41] Lau CE Tredwell GD Ellis JK Lam EW Keun HC. 'Metabolomic Characterisation of the Effects of Oncogenic PIK3CA Transformation in a Breast Epithelial Cell Line'. Sci Rep (2017) 7:46079. doi: 10.1038/srep46079 28393905 PMC5385542

[42] Uchida K Sun W Yamazaki J Tominaga M. 'Role of Thermo-Sensitive Transient Receptor Potential Channels in Brown Adipose Tissue'. Biol Pharm Bull (2018) 41:1135–44. doi: 10.1248/bpb.b18-00063 30068861

[43] Sun W Yu Z Yang S Jiang C Kou Y Xiao L. 'A Transcriptomic Analysis Reveals Novel Patterns of Gene Expression During 3t3-L1 Adipocyte Differentiation'. Front Mol Biosci (2020) 7:564339. doi: 10.3389/fmolb.2020.564339 33195411 PMC7525235

[44] Laffita-Mesa JM Nennesmo I Paucar M Svenningsson P. 'A Novel Duplication in ATXN2 as Modifier for Spinocerebellar Ataxia 3 (SCA3) and C9ORF72-Als'. Mov Disord (2021) 36:508–14. doi: 10.1002/mds.28334 PMC7983901 33058338

[45] Song XH He N Xing YT Jin XQ Li YW Liu SS. 'A Novel Age-Related Circular RNA Circ-ATXN2 Inhibits Proliferation, Promotes Cell Death and Adipogenesis in Rat Adipose Tissue-Derived Stromal Cells'. Front Genet (2021) 12:761926. doi: 10.3389/fgene.2021.761926 34858478 PMC8630790

[46] Parks JS Chung S Shelness GS. 'Hepatic ABC Transporters and Triglyceride Metabolism'. Curr Opin Lipidol (2012) 23:196–200. doi: 10.1097/MOL.0b013e328352dd1a 22488425 PMC3793202

[47] Ma D Liu W Wang Y. 'Apoa-I or ABCA1 Expression Suppresses Fatty Acid Synthesis by Reducing 27-Hydroxycholesterol Levels'. Biochimie (2014) 103:101–8. doi: 10.1016/j.biochi.2014.04.010 24793484

[48] Qiu J Wu B Goodman SB Berry GJ Goronzy JJ Weyand CM. 'Metabolic Control of Autoimmunity and Tissue Inflammation in Rheumatoid Arthritis'. Front Immunol (2021) 12:652771. doi: 10.3389/fimmu.2021.652771 33868292 PMC8050350

[49] Yates CM Calder PC Ed Rainger G. 'Pharmacology and Therapeutics of Omega-3 Polyunsaturated Fatty Acids in Chronic Inflammatory Disease'. Pharmacol Ther (2014) 141:272–82. doi: 10.1016/j.pharmthera.2013.10.010 24201219

[50] Yang L Guan G Lei L Lv Q Liu S Zhan X. 'Palmitic Acid Induces Human Osteoblast-Like Saos-2 Cell Apoptosis *via* Endoplasmic Reticulum Stress and Autophagy'. Cell Stress Chaperones (2018) 23:1283–94. doi: 10.1007/s12192-018-0936-8 PMC6237680 30194633

[51] Wallace M Metallo CM. 'Tracing Insights Into *De Novo* Lipogenesis in Liver and Adipose Tissues'. Semin Cell Dev Biol (2020) 108:65–71. doi: 10.1016/j.semcdb.2020.02.012 32201132

[52] Chen X Xun K Chen L Wang Y. 'TNF-Alpha, a Potent Lipid Metabolism Regulator'. Cell Biochem Funct (2009) 27:407–16. doi: 10.1002/cbf.1596 19757404

[53] Xu Y Zhang Y Ye J. 'Il-6: A Potential Role in Cardiac Metabolic Homeostasis'. Int J Mol Sci (2018) 19(9):2474. doi: 10.3390/ijms19092474 PMC6164544 30134607

[54] Kawase A Yoshida I Tsunokuni Y Iwaki M. 'Decreased PXR and CAR Inhibit Transporter and CYP mRNA Levels in the Liver and Intestine of Mice With Collagen-Induced Arthritis'. Xenobiotica (2007) 37:366–74. doi: 10.1080/00498250701230534 17455111

[55] Sundaram MS Neog MK Rasool M Kumar GS Hemshekhar M Kemparaju K. 'Guggulipid Ameliorates Adjuvant-Induced Arthritis and Liver Oxidative Damage by Suppressing Inflammatory and Oxidative Stress Mediators'. Phytomedicine (2019) 64:152924. doi: 10.1016/j.phymed.2019.152924 31465983

[56] Sukketsiri W Chonpathompikunlert P Tanasawet S Choosri N Wongtawatchai T. 'Effects of Apium Graveolens Extract on the Oxidative Stress in the Liver of Adjuvant-Induced Arthritic Rats'. Prev Nutr Food Sci (2016) 21:79–84. doi: 10.3746/pnf.2016.21.2.79 27390722 PMC4935245

[57] Zhang T Gillies MC Madigan MC Shen W Du J Grünert U. 'Disruption of *De Novo* Serine Synthesis in Müller Cells Induced Mitochondrial Dysfunction and Aggravated Oxidative Damage'. Mol Neurobiol (2018) 55:7025–37. doi: 10.1007/s12035-017-0840-8 29383682

[58] Wang R Tang R Li B Ma X Schnabl B Tilg H. 'Gut Microbiome, Liver Immunology, and Liver Diseases'. Cell Mol Immunol (2021) 18:4–17. doi: 10.1038/s41423-020-00592-6 33318628 PMC7852541

[59] Yashin DV Romanova EA Ivanova OK Sashchenko LP. 'The Tag7-Hsp70 Cytotoxic Complex Induces Tumor Cell Necroptosis *via* Permeabilisation of Lysosomes and Mitochondria'. Biochimie (2016) 123:32–6. doi: 10.1016/j.biochi.2016.01.007 26796882

[60] Luo Q Li X Zhang L Yao F Deng Z Qing C. 'Serum PGLYRP−1 is a Highly Discriminatory Biomarker for the Diagnosis of Rheumatoid Arthritis'. Mol Med Rep (2019) 19:589–94. doi: 10.3892/mmr.2018.9632 30431075

[61] Yong L Guang B Yan L. 'Bioinformatic Analysis of Differentially Expressed Genes Involved in the Hepatitis B Virus-Associated Acute Liver Failure'. Acta Gastroenterol Belg (2018) 81:288–94. 30024701

[62] Cheng X Liu D Song H Tian X Yan C Han Y. 'Overexpression of Kininogen-1 Aggravates Oxidative Stress and Mitochondrial Dysfunction in DOX-Induced Cardiotoxicity'. Biochem Biophys Res Commun (2021) 550:142–50. doi: 10.1016/j.bbrc.2021.02.104 33706097

[63] Singh S Vennila JJ Snijesh VP George G Sunny C. 'Implying Analytic Measures for Unravelling Rheumatoid Arthritis Significant Proteins Through Drug-Target Interaction'. Interdiscip Sci (2016) 8:122–31. doi: 10.1007/s12539-015-0108-9 26286007

[64] Zhang Q He Y Luo N Patel SJ Han Y Gao R. 'Landscape and Dynamics of Single Immune Cells in Hepatocellular Carcinoma'. Cell (2019) 179:829–45.e20. doi: 10.1016/j.cell.2019.10.003 31675496

[65] Tian F Liu C Wu Q Qu K Wang R Wei J. 'Upregulation of Glycoprotein Nonmetastatic B by Colony-Stimulating Factor-1 and Epithelial Cell Adhesion Molecule in Hepatocellular Carcinoma Cells'. Oncol Res (2013) 20:341–50. doi: 10.3727/096504013X13657689382851 23924854

[66] Tsou PS Sawalha AH. 'Glycoprotein Nonmetastatic Melanoma Protein B: A Key Mediator and an Emerging Therapeutic Target in Autoimmune Diseases'. FASEB J (2020) 34:8810–23. doi: 10.1096/fj.202000651 PMC7501235 32445534

[67] Zohar Y Wildbaum G Karin N. 'Beneficial Autoimmunity Participates in the Regulation of Rheumatoid Arthritis'. Front Biosci (2006) 11:368–79. doi: 10.2741/1804 16146738

[68] Zhebrun DA Totolyan AA Maslyanskii AL Titov AG Patrukhin AP Kostareva AA. 'Synthesis of Some CC Chemokines and Their Receptors in the Synovium in Rheumatoid Arthritis'. Bull Exp Biol Med (2014) 158:192–6. doi: 10.1007/s10517-014-2720-9 25430645

[69] Miellot-Gafsou A Biton J Bourgeois E Herbelin A Boissier MC Bessis N. 'Early Activation of Invariant Natural Killer T Cells in a Rheumatoid Arthritis Model and Application to Disease Treatment'. Immunology (2010) 130:296–306. doi: 10.1111/j.1365-2567.2009.03235.x 20113367 PMC2878473

[70] Kakinuma Y Kimura T Watanabe Y. 'Possible Involvement of Liver Resident Macrophages (Kupffer Cells) in the Pathogenesis of Both Intrahepatic and Extrahepatic Inflammation'. Can J Gastroenterol Hepatol (2017) 2017:2896809. doi: 10.1155/2017/2896809 28804705 PMC5539927

[71] Romano C Del Mastro A Sellitto A Solaro E Esposito S Cuomo G. 'Tocilizumab Reduces Complement C3 and C4 Serum Levels in Rheumatoid Arthritis Patients'. Clin Rheumatol (2018) 37:1695–700. doi: 10.1007/s10067-018-3992-7 29362962

[72] Sena L Oliveira-Tore CF Skare T de Messias-Reason IJ Andrade FA. 'C3 Gene Functional Polymorphisms and C3 Serum Levels in Patients With Rheumatoid Arthritis'. Immunol Invest (2021) 50:1027–41. doi: 10.1080/08820139.2020.1800726 32787514

[73] Dejonckheere E Vandenbroucke RE Libert C. 'Matrix Metalloproteinase8 has a Central Role in Inflammatory Disorders and Cancer Progression'. Cytokine Growth Factor Rev (2011) 22:73–81. doi: 10.1016/j.cytogfr.2011.02.002 21388856

[74] Yoshihara Y Nakamura H Obata K Yamada H Hayakawa T Fujikawa K. 'Matrix Metalloproteinases and Tissue Inhibitors of Metalloproteinases in Synovial Fluids From Patients With Rheumatoid Arthritis or Osteoarthritis'. Ann Rheum Dis (2000) 59:455–61. doi: 10.1136/ard.59.6.455 PMC1753174 10834863

[75] Akhavani MA Madden L Buysschaert I Sivakumar B Kang N Paleolog EM. 'Hypoxia Upregulates Angiogenesis and Synovial Cell Migration in Rheumatoid Arthritis'. Arthritis Res Ther (2009) 11:R64. doi: 10.1186/ar2689 19426483 PMC2714109

[76] Kos J Jevnikar Z Obermajer N. 'The Role of Cathepsin X in Cell Signaling'. Cell Adh Migr (2009) 3:164–6. doi: 10.4161/cam.3.2.7403 PMC2679876 19262176

[77] Lechner AM Assfalg-Machleidt I Zahler S Stoeckelhuber M Machleidt W Jochum M. 'RGD-Dependent Binding of Procathepsin X to Integrin Alphavbeta3 Mediates Cell-Adhesive Properties'. J Biol Chem (2006) 281:39588–97. doi: 10.1074/jbc.M513439200 17065156

[78] de Seny D Baiwir D Bianchi E Cobraiville G Deroyer C Poulet C. 'New Proteins Contributing to Immune Cell Infiltration and Pannus Formation of Synovial Membrane From Arthritis Diseases'. Int J Mol Sci (2021) 23(1):434. doi: 10.3390/ijms23010434. 35008858 PMC8745719

[79] Simard JC Girard D Tessier PA. 'Induction of Neutrophil Degranulation by S100A9 *via* a MAPK-Dependent Mechanism'. J Leukoc Biol (2010) 87:905–14. doi: 10.1189/jlb.1009676 20103766

[80] Kang KY Woo JW Park SH. 'S100a8/A9 as a Biomarker for Synovial Inflammation and Joint Damage in Patients With Rheumatoid Arthritis'. Korean J Intern Med (2014) 29:12–9. doi: 10.3904/kjim.2014.29.1.12 PMC3932383 24574827

