# Supplementary material for: Analysis of Hepatic Lipid Metabolism and Immune Function During the Development of Collagen-Induced Arthritis
Source: Front Immunol. 2022 Jun 16;13:901697. doi: 10.3389/fimmu.2022.901697 (PMC9245434; doi:10.3389/fimmu.2022.901697)
Supplement: Supplementary file 1 [file Table_1.docx]

Supplementary Material

## Supplementary Tables

**Supplementary Table 1. A list of the targeted FAs in this study**

| FAs | quantification ion pair(m/z) | Fragment  Voltage (V) | Collision  Energy (eV) |
| --- | --- | --- | --- |
| C12:0 | 199.2＞199.2 | 70 | 20 |
| C12:1 | 197.1＞197.1 | 80 | 5 |
| C14:0 | 227.2＞227.2 | 90 | 20 |
| C14:1 | 225.2＞225.2 | 120 | 10 |
| C15:0 | 241.2＞241.2 | 90 | 0 |
| C16:0 | 255.2＞255.2 | 90 | 5 |
| C16:1 | 253.2＞253.2 | 120 | 5 |
| C17:0 | 269.3＞269.3 | 70 | 5 |
| C18:0 | 283.2＞283.2 | 80 | 20 |
| C18:1 | 281＞281 | 120 | 0 |
| C18:2 | 279.2＞279.2 | 90 | 10 |
| C18:3 | 277.2＞277.2 | 70 | 0 |
| C20:0 | 311.2＞311.2 | 70 | 5 |
| C20:4 | 303.3＞303.2 | 60 | 0 |
| C20:5 | 301.2＞257.3 | 120 | 0 |
| C22:0 | 339.3＞339.3 | 100 | 20 |
| C22:2 | 337.3＞ 337.4 | 100 | 5 |
| C22:6 | 327.3＞327.3 | 100 | 5 |

**Supplementary Table 2. Lipid metabolism related function annotation in differential phases**

| Phase | Lipid metabolism related function annotation | -log(p-value) |
| --- | --- | --- |
| Induction | Concentration of lipid | 4.63 |
| Induction | Synthesis of lipid | 3.11 |
| Induction | Metabolism of membrane lipid derivative | 3.98 |
| Induction | Concentration of triacylglycerol | 4.43 |
| Induction | Quantity of steroid | 2.47 |
| Induction | Concentration of phospholipid | 4.96 |
| Induction | Concentration of sterol | 2.82 |
| Induction | Synthesis of phospholipid | 3.52 |
| Induction | Transport of lipid | 3.47 |
| Induction | Concentration of cholesterol | 2.57 |
| Induction | Release of lipid | 3.15 |
| Induction | Synthesis of phosphatidic acid | 2.55 |
| Induction | Abnormal quantity of lipid | 3.65 |
| Induction | Release of eicosanoid | 3.49 |
| Induction | Transport of sterol | 3.33 |
| Induction | Efflux of lipid | 2.99 |
| Induction | Cholesterol transport | 2.77 |
| Induction | Transport of phospholipid | 4.45 |
| Induction | Efflux of cholesterol | 2.63 |
| Induction | Metabolism of cholesterol | 2.46 |
| Induction | Clearance of triacylglycerol | 5.47 |
| Induction | Translocation of phospholipid | 4.84 |
| Induction | Accumulation of cholesterol | 2.63 |
| Induction | Abnormal quantity of phospholipid | 3.64 |
| Induction | Quantity of phosphatidylinositol phosphate | 3.37 |
| Induction | Internalization of lipid | 3.31 |
| Induction | Delay in clearance of lipid | 5.68 |
| Induction | Uptake of cholesterol ester | 3.16 |
| Induction | Import of lipid | 2.83 |
| Induction | Quantity of C24:1-ceramide | 2.76 |
| Induction | Release of leukotriene C4 | 2.41 |
| Induction | Delay in clearance of triacylglycerol | 3.79 |
| Induction | Recycling of lactosylceramide | 3.79 |
| Induction | Import of GABA | 3.01 |
| Induction | Transport of cholesterol ester | 2.48 |
| Peak | Concentration of lipopolysaccharide | 3.07 |
| Peak | Metabolism of triacylglycerol | 2.76 |
| Resolution | Hydroxylation of pregnenolone | 4.48 |
| Resolution | Efflux of cholesterol | 3.76 |
| Resolution | Reverse cholesterol transport | 3.51 |

**Supplementary Table 3. The Log2(fold change) and p-value of DEPs which were verified in lipid metabolism network in the induction phase**

| DEPs | Log2(fold change) | p-value |
| --- | --- | --- |
| TRPM4 | 6.64 | 0.047 |
| ATXN2 | 3.81 | 0.044 |
| PIK3C2a | 3.48 | 0.037 |

**Supplementary Table 4. The Log2(fold change) and p-value of DEPs which were verified in immune function network at the peak phase**

| DEPs | Log2(fold change) | p-value |
| --- | --- | --- |
| PGLYRP1 | 6.64 | 0.017 |
| GPNMB | 6.64 | 0.031 |
| KNG1 | 3.62 | 0.002 |
